# Prevalence of menopausal symptoms among mid-life women: findings from electronic medical records

**DOI:** 10.1186/s12905-015-0217-y

**Published:** 2015-08-13

**Authors:** Matthew Sussman, Jeffrey Trocio, Craig Best, Sebastian Mirkin, Andrew G. Bushmakin, Robert Yood, Mark Friedman, Joseph Menzin, Michael Louie

**Affiliations:** Boston Health Economics, Inc., 20 Fox Road, Waltham, MA 02451 USA; Pfizer Inc., 235 East 42nd Street, New York, NY 10017 USA; Reliant Medical Group, 630 Plantation Street, Worcester, MA 01605 USA

## Abstract

**Background:**

To assess the prevalence of menopausal symptoms among women prescribed hormone therapy (HT) using electronic medical record data from a regional healthcare organization.

**Methods:**

Retrospective data from the Reliant Medical Group from 1/1/2006-12/31/2011 were assessed for 102 randomly-selected patients. Study eligibility criteria included: females aged 45 to 65; prescribed oral or transdermal HT; no history of breast cancer, venous thromboembolism, stroke, gynecological cancer, or hysterectomy; continuously enrolled in the health plan for 1 year before and after the first observed HT prescription. Prevalence of menopause-related symptoms was analyzed descriptively at both the patient and visit levels.

**Results:**

Mean age of patients was 54 years. The most common menopausal symptoms were: hot flushes (40 %), night sweats (17 %), insomnia (16 %), vaginal dryness (13 %), mood disorders (12 %), and weight gain (12 %). Among the 102 patients, 163 individual visits listing menopausal symptoms were identified, of which hot flushes (71 visits) were the most common symptom identified.

**Conclusion:**

Our findings provide recent data on the types of menopausal symptoms experienced by mid-life women prescribed HT. Electronic medical records may be a rich source of data for future studies of menopausal symptoms in this population.

## Background

Menopause, defined as the complete cessation of menstrual periods, occurs naturally in most women and is associated with the gradual loss of ovarian follicles. With the aging of the worldwide population in the coming decades, it is estimated that 1.2 billion women worldwide will be menopausal or postmenopausal by the year 2030 [1]. Common symptoms of menopause include vasomotor symptoms (VMS, defined as hot flushes and/or night sweats), sleep disturbances, and vaginal dryness [2]. It is estimated that as many as 85 % of postmenopausal women have experienced a menopause-related symptom in their lifetime [3]. Prevalence of VMS alone is estimated at approximately 40 to 50 million women in the United States [4].

Symptom prevalence and severity generally increase with advancing reproductive stage, which ranges from late reproductive, early menopause transition, late menopause transition, to postmenopause [3]. Currently, therapies targeted to treat a variety of menopausal symptoms are hormone-based. These therapies are generally more effective than non-hormonal treatments which generally treat a single symptom. However, hormone therapy (HT) is not indicated for women with past or existing breast cancer, past or existing estrogen-sensitive malignant conditions, undiagnosed genital bleeding, untreated endometrial hyperplasia, venous thromboembolic event history, past or existing arterial thromboembolic disease, untreated hypertension, existing liver disease, hypersensitivity to the active ingredients in HT, and porphyria cutanea tarda [5]. Current guidelines recommend that HT is prescribed at the lowest effective dose and for the shortest duration, and be consistent with individual treatment goals among postmenopausal women [6].

The primary objective of this study was to assess the prevalence of menopausal symptom burden among women prescribed HT using electronic medical record (EMR) data. EMR systems are an emerging, rich data source for capturing detailed information during real-world medical encounters, and thus were the primary vehicle for this analysis.

## Methods

### Overview

This study evaluated the prevalence and burden of menopause-related symptoms among women aged 45 to 65 who had evidence of HT use. EMR data from a regional healthcare organization were analyzed for prevalence of menopausal symptoms. Women were selected for potential inclusion if they were enrolled in the Fallon Community Health Plan for one year prior to and one year following the first observed prescription for HT.

### Data source

Data from the Reliant Medical Group, Worcester, Massachusetts, USA were used to explore the prevalence of menopausal symptoms among women undergoing HT. The Reliant Medical Group is a multispecialty group practice with a predominantly managed-care population of approximately 200,000 patients. Data covering the six-year period from January 1, 2006 through December 31, 2011 (the study period) were analyzed.

The study data set consisted of combined patient medical claims and EMR data. Patients’ data were de-identified, with a unique, encrypted identifier available to link claims and EMR information. The study protocol and data collection forms were approved by the Reliant Medical Group-Fallon Community Health Plan-Saint Vincent Hospital Institutional Review Board.

### Patient selection

Patient IDs for EMR review were identified based on a filled prescription for HT, using administrative claims data from the Reliant Medical Group. Patients selected for study inclusion were first identified using the following claims-based eligibility criteria: female patients, fill of at least one month’s supply of HT anytime between January 1, 2007 and December 31, 2010 (with the first date of HT deemed the “index date” for analysis), and aged 45 to 65 years of age as of the index date. Patients were additionally required to have no history of breast cancer, venous thromboembolism, stroke, gynecological cancer, or total hysterectomy, and be continuously enrolled in the health plan for 12 months pre- and 12 months post-index. HT consisted of oral or transdermal estrogen or estrogen and progestin combination therapies, excluding HT rings and creams.

From the full list of patients meeting eligibility criteria in claims data, a random sample of patients was generated to serve as the cohort for the EMR review. Study inclusion criteria, as first identified via claims data, were confirmed through the EMR chart review by clinical professionals from the Reliant Medical Group until approximately 100 patients met eligibility criteria, representing approximately one-third of patients meeting claims eligibility criteria.

### Menopausal symptom identification

All patient visits in the EMR (excluding behavioral management visits) occurring within the two-year study period were analyzed. Data variables extracted from the EMR data set included: visit date, reason for the visit, provider specialty, and whether any menopausal symptoms were listed during the visit. All symptoms were required to have menopause referenced in the physician notes of the EMR as the cause of the symptom. Symptoms could have been self-reported by the patient or elicited by the healthcare provider. Specific menopause symptoms evaluated included: hot flushes (used interchangeably with hot flashes), night sweats, insomnia, vaginal dryness, loss of sexual desire, weight gain, hair loss, fatigue, major depression, anxiety, or mood disorders (including mention of episodic mood disorder and/or mood swings). VMS was defined based on the presence of hot flushes and/or night sweats, which is consistent with the National Institute of Health (NIH) definition of VMS [7].

### Data analysis

The prevalence of all identified symptoms was analyzed descriptively at both the patient and visit levels. Descriptive analyses of all patient characteristics were conducted. Binary variables were summarized using percentages of patients and continuous variables were summarized using mean, SD, and median values. All analyses were conducted with SAS v9.3, Cary, North Carolina, USA.

## Results

### Patient characteristics

A total of 102 patients were randomly selected from the administrative claims data and met inclusion criteria for full EMR chart review. The highest proportion of patients was between 50-54 years of age (41 %), while 33 % were aged 55-59. Overall, the mean (±SD) age was 54 (±5) years. Approximately one-third of patients were categorized as being normal weight (32 %), overweight (34 %), or obese/morbidly obese (29 %) (Table 1).

**Table 1:** Demographic characteristics of patient sample

Characteristic	Estimate
Patients (N)	102
Age (%)	
45 - 49	11.8 %
50 - 54	41.2 %
55 - 59	33.3 %
60 - 65	13.7 %
Mean	54
SD	5
Median	54
Height as of index date (inches)	
Mean	63.9
SD	2.3
Median	64.0
Weight as of index date (pounds)	
Mean	163.7
SD	36.6
Median	158.0
Missing (%)	1.0 %
Body mass index	
Mean	28.1
SD	6.4
Median	26.9
Obesity status (%)^a^	
Underweight	2.0 %
Normal	32.4 %
Overweight	34.3 %
Obese	24.5 %
Morbidly obese	4.9 %
Missing (%)	2.0 %

Index was defined as the date of the first HT use

^a^Underweight: BMI <18.5; Normal: 18.5 ≤ BMI to < 25; Overweight: 25 ≤ BMI < 30; Obese: 30 ≤ BMI < 40; Morbidly Obese: BMI ≥ 40

### Menopausal symptom identification

**Fig. 1:**
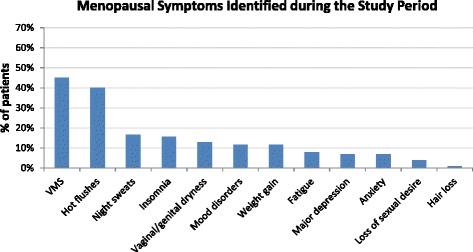
Percent of patients with a diagnosis of select menopausal symptoms over a two-year analysis period

At the patient level, the most common menopausal symptoms identified during the study period using EMR data were: hot flushes (40 %), followed by night sweats (17 %), insomnia (16 %), vaginal dryness (13 %), mood disorders (12 %), and weight gain (12 %) (Fig. 1). 45 % of the patients presented with VMS (either hot flushes or night sweats).

Among the 102 patients included in this analysis, a total of 163 individual visits listed menopausal symptoms during the study period. Similar to the patient-level analysis, the most common symptom identified in visit-level EMR data were hot flushes (71 visits, 44 % of individual visits), the estimates for which indicate that some patients had more than one complaint.

### Provider specialty

For visits with any menopausal symptom identified, the highest proportion of patients were seen by an OB/GYN (46 %), followed by 36 % for internal medicine, 10 % for family practitioner, and 8 % for other specialty type. More specifically, the vast majority of hot flushes and night sweats were identified by either an OB/GYN or an internal medicine physician.

## Discussion

### Summary

This analysis examined menopausal symptoms among 102 midlife women in a Massachusetts-based healthcare organization. Results from this analysis provide recent data on the types of menopausal symptoms experienced among midlife women prescribed HT. Approximately half of women were found to experience hot flushes.

### Comparison to literature

Following a comprehensive search of the literature, we were unable to find any published studies using EMR data to assess menopausal symptom burden. Studies using alternative methodologies to assess the prevalence of various menopausal symptoms exist [8–11]; however, it is difficult to directly compare their results to those from our study.

For instance, two studies [8, 9] used survey-based methodology to determine prevalence rates of menopausal symptoms, yet this technique does not represent real-world practice as patients may be more willing to disclose sensitive symptoms given the private format of administering and completing surveys. Another study used face-to-face interviews to collect data on menopausal symptoms [10]; however, not all patients in the study were required to be on HT, therefore limiting the comparability to our population of women prescribed HT. Conducting survey-based research is expensive and time consuming, due to efforts to design the survey, to prepare documents for institutional review board approval, to administer the survey, and to collect and enter data for analysis. EMR data, in contrast, offers the benefit of providing ready access to up-to-date information and is an emerging source for patient data given the proliferation of EMR systems in recent years [12].

Menopausal symptom burden was also assessed in the context of a prospective observational study, which included follow-up visits to specifically evaluate rates of menopausal symptoms. Follow-up focused on menopausal symptoms likely led to higher prevalence estimates than what would be expected to be captured in real-world practice such as our study [11].

Finally, we were unable to identify any studies that used administrative claims data to assess our research objectives. There are a variety of reasons of why this may be the case. While accurate in identifying many common diagnoses, administrative claims data are subject to medical coding errors since not all services are billed [13]. Diagnoses recorded in claims may be limited to a select few diagnoses associated with an office visit, as opposed to the more complete set of information available in physician notes in an EMR system. This discrepancy in diagnoses may be of particular importance among women treated for menopause with HT, where an annual visit to the gynecologist or primary care provider may be simply listed in claims as a general medical examination, rather than including the full set of symptoms discussed during the visit.

### Limitations

The application of EMR data to this analysis carries certain limitations which may have contributed to an underestimation of symptom prevalence. For instance, the EMR analysis relied on patients to report relevant information regarding menopausal symptoms to their healthcare providers. For symptoms deemed to be highly sensitive, patients may have chosen not to report such symptoms to their providers. Similarly, the symptoms recorded in the EMR system were at the discretion of the attending healthcare provider. It is possible that some complaints deemed to be non-severe and/or not requiring treatment were not recorded in the EMR. It is also important to note that the severity of symptoms could not be identified based on data extracted from the EMR. For patients who were seen at providers outside of the Reliant Medical Group, symptoms would not have been captured in the EMR system. Additionally, symptoms not directly documented by the physician as menopause-related were not analyzed in the EMR data. Moreover, the duration of HT use was not captured in the data set, which may have had an impact on the prevalence and/or severity of menopausal symptoms. Finally, the data set analyzed in the present study was limited to patients enrolled in the Fallon Community Health Plan which covers only the Massachusetts area, and therefore does not provide nationally-representative estimates.

## Conclusion

This study confirmed that EMR data may be a rich source of information regarding menopausal symptoms among women prescribed HT. Our study provides current estimates of the symptom burden among women undergoing HT, with approximately half of patients reporting VMS in clinical practice. As the use of EMR systems grow, these data may become increasingly useful as a source of patient-reported outcomes information.
